# 8000-year monsoonal record from Himalaya revealing reinforcement of tropical and global climate systems since mid-Holocene

**DOI:** 10.1038/s41598-017-15143-9

**Published:** 2017-11-06

**Authors:** Pradeep Srivastava, Rajesh Agnihotri, Deepti Sharma, Narendra Meena, Y. P. Sundriyal, Anju Saxena, Ravi Bhushan, R. Sawlani, Upasana S. Banerji, C. Sharma, P. Bisht, N. Rana, R. Jayangondaperumal

**Affiliations:** 1 Wadia Institute of Himalayan Geology, Dehradun, India; 2 Birbal Sahni Institute of Palaeosciences, Lucknow, India; 3 HNB Garhwal University, Srinagar, Uttarakhand India; 4 Physical Research Laboratory, Ahmadabad, India; 5 CSIR- National Physical Laboratory, New Delhi, India

## Abstract

We provide the first continuous Indian Summer Monsoon (ISM) climate record for the higher Himalayas (Kedarnath, India) by analyzing a ^14^C-dated peat sequence covering the last ~8000 years, with ~50 years temporal resolution. The ISM variability inferred using various proxies reveal striking similarity with the Greenland ice core (GISP2) temperature record and rapid denitrification changes recorded in the sediments off Peru. The Kedarnath record provides compelling evidence for a reorganization of the global climate system taking place at ~5.5 ka BP possibly after sea level stabilization and the advent of inter-annual climate variability governed by the modern ENSO phenomenon. The ISM record also captures warm-wet and cold-dry conditions during the Medieval Climate Anomaly and Little Ice Age, respectively.

## Introduction

The Himalaya is the tallest tectonically-uplifted mountain range in the Indo-Pakistan-China region, currently supporting ~15% of the world’s population^1^. Several past human civilizations have originated, flourished, and disappeared along the flow paths of major Himalayan river systems, including the Indus-Saraswati, and Ganga-Yamuna-Brahmaputra. Precipitation associated with the Indian Summer Monsoon, melting of glaciers, and sub-surface (ground) aquifers collectively ensure the availability of water in these river systems. Additionally, high-altitude regions of Himalaya also receive rainfall induced by westerly winds during late winter and spring seasons. In the scenario of global warming, the duration of monsoonal-rainfall is anticipated to decrease but the frequency of extreme rainfall events may increase^2^. The concurrent precipitation trends appear to be supportive of such a case^3^. For example, recent rainfall events, such as in 2013 (mid-June) when massive flash floods caused colossal damage to human-life and infrastructure worth ~$3 billion^4^, indicate emergence of such a scenario. Recent studies strongly indicate that a above normal monsoon year is likely to be punctuated with high rainfall events^3,5^. Reconstruction of high-resolution monsoonal variability spanning the Holocene is required to better understand the changing rainfall patterns amidst anthropogenic climate change^6^. The Kedarnath area in the Garhwal Himalaya could serve as an excellent locale for recovering such paleo-records. As the Kedarnath area lies over the northern limits of Inter Tropical Convergence Zone (ITCZ), it is expected to capture subtle changes in the ISM variability.

To date, the majority of the ISM records spanning the Holocene are from Arabian Sea sediments, cave deposits from peninsular India and lacustrine/peat sequences from central and north-western India^7–17^. Retrieving high-resolution monsoonal variability from terrestrial geological records is complicated due to several reasons such as regional influences masking the climatic signal, variable proxy response, and chronological uncertainties. Nonetheless, a few paleo-monsoonal records covering the Holocene have recently been reconstructed from the northwest Himalaya^13,15,18,19^. A globally inter-comparable ISM record depicting Holocene climate variability, however, remains elusive. Monsoon climate experts and modelers underscore the importance of reliable proxy-records dating back to ~6 ka BP when sea levels stablized globally^20^. These records can be exploited to ascertain natural climate variability and primary operative forcing factors.

To fill this important knowledge-gap, we present here a multi-proxy palaeo-monsoonal record from the Kedarnath, Uttarakhand, India (30.73°N; 79.07°E). The study-site is located at an altitude of ~3525 meters above mean sea level (AMSL). Chronology of 5.22 meter long peat-sequence was obtained from seven ^14^C dates of organic carbon measured by Accelerator Mass Spectrometry (AMS). Sampling intervals and identified chronology enabled us to decipher proxy variations mimicking past monsoon variability with a ~50-year temporal resolution. Remarkable concurrences were found among different proxies, such as chemical composition (element abundances), stable isotopic anomalies, magnetic mineral susceptibility, and palynological assemblages. The identified ISM record reveals striking similarities with the northern hemispheric temperature variations recorded in the Greenland Ice core (GISP2) and the multi-decadal scale denitrification changes retrieved from sedimentary records off Peru. Worthy to mention the Peru margin area is the heartland of El Niño Southern Oscillations (ENSO), which is known to impact global, as well as Indian monsoonal climate, significantly. Noticeably higher and lower monsoonal strengths could be inferred during the Medieval Climate Anomaly (MCA) and the Little Ice Age (LIA), respectively. In addition, a high-frequency variability in monsoonal intentisty is recorded between ~5.4 to ~3.8 ka BP, which overlapps with several pre-historic human-settlements: *e.g*. Indus (Harappan) and the Ganga valley civilizations.

## Climate, History, and Vegetation of the Study Location

**Figure 1:**
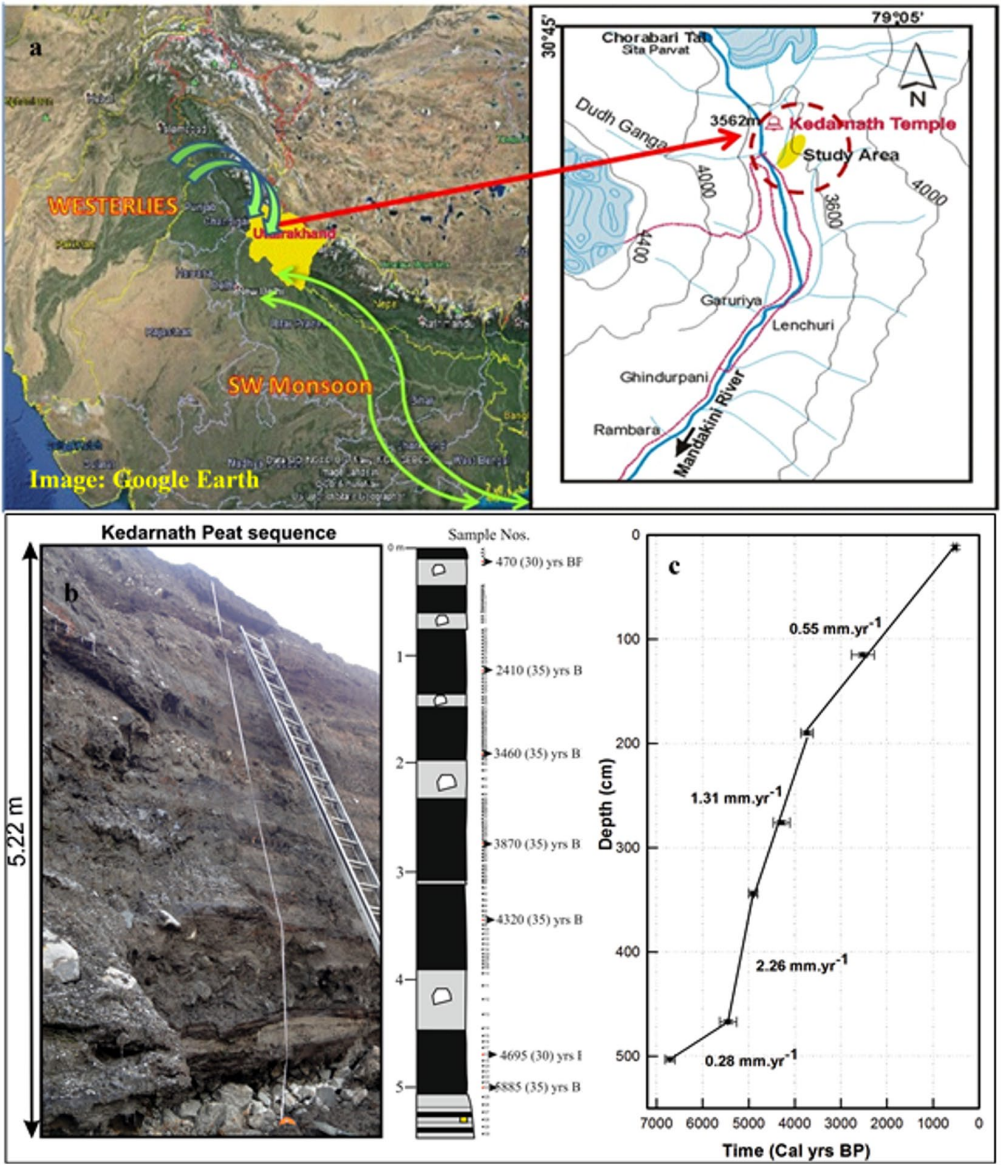
(**a**) Location, altitudinal map and photograph of the Kedarnath peat sequence. (**b**) Field photograph and litholog (panel ‘b’) were made using the MS-Coral Draw-X6c). Litholog is juxtaposed with sample locations of the studied peat sequence taken for AMS radiocarbon dates. (**c**) The age-depth model constructed using seven AMS radiocarbon dates using licensed version of sigma plot (v.10). Satellite image frame used in ‘panel a’ is adopted from Google Earth (version freely available for Windows 7).

The peat-sequence is located in the vicinity of Kedarnath Temple, a famous Hindu shrine since pre-historic times^21^. As evident from the Fig. 1, the peat-sequence accumulated in the depression between the two moraine ridges (dated to between ~13 and ~7 ka; Fig. S1)^22^ and is located ~340 m below the snout of the Chorabari Glacier (~3500 m AMSL; N30.73°, E79.06°).The sequence is composed of several units of bedded fibrous peat with alternating layers of black colored sandy mud with sporadic angular gravels. A total of 129 samples were collected from different units, with sampling interval varying from 3–10 cm. Rainfall data from the weather station installed near the snout of the Chorabari Glacier and Tropical Radar Rainfall Measurement Mission (TRMM) product reveal that the annual rainfall in region is dominated by ISM (see Supplementary Fig. S2). The time-series data of all India annual average rainfall and summer monsoon rainfall (June, July, August and September; JJAS for 1951–2000 AD) show significant statistical correlation (0.5–0.6; p = 0.01) with that falling over the state of Uttarakhand (see Supplementary Fig. S3). The Kedarnath area falls on the northern periphery of ISM coverage *i.e*. the farthest reach of monsoonal rainfall that originates via differential heating of continental and marine realms of Indian sub-continent and northern Indian Ocean, respectively. Therefore, delicate and subtle changes in primary and secondary forcing factors responsible for net ISM performance are expected to be well-preserved in the geological archives of the region.

Winter precipitation at the site is mainly composed of snow that falls between the months of December and March, under dominance of westerly-winds. The ambient summer temperatures typically vary from −1 to +17 °C (between June to October); whereas winter-temperature may plunge to ~−20 °C. The entire Kedarnath region is characterized by its rich-biodiversity and varying topographic features (*e.g*., altitudinal ranges, slope, gradient), where climate (rainfall pattern, wind-circulation) is controlled by several edaphic factors. The surface vegetation mainly consists of various forest elements, alpine meadows, and scrubby elements. The arboreal forest vegetation is dominated by conifers with subordinate broad-leave tree species^23^.

## Chronology and Age-Depth Model

For ascertaining chronology of the peat-sequence, seven bulk samples (see Fig. 1) were chosen for ^14^C dating by Accelerator Mass Spectrometry (AMS) which was carried out at the Poznan Radiocarbon Laboratory, Poland. All ^14^C ages were calibrated using the online OxCal program (version 4.1.7)^24^ which uses the IntCal13 dataset^25^. The calibrated ages are presented in Fig. 1 and Table S1. All ^14^C ages are presented with 2σ uncertainty (reflecting 95.4% probability) (see Table S1). Figure 1c shows the adopted age-depth model revealing variable rates of peat-formation. For the last ~3.7 ka, the peat-deposition rate is estimated to be 0.55 mm.a^−1^.Between ~3.7 to 4.9 ka BP and ~4.9 to 5.5 ka, deposition rates were relatively higher ~1.3 and 2.26 mm.a^−1^, respectively. Thereafter, again the peat deposition rate slowed down (~0.28 mm.a^−1^; Fig. 1c). Maximum uncertainties associated with the calibrated radiocarbon ages were ~200 years (2σ) (Table S1); and overall uncertainty due to age–depth model adopted could vary between ~200–320 years (considering uncertainties with the standard-fits).

## Proxies and Interpretative Framework

Snow-melt and direct monsoonal precipitation govern regional hydrology, where ambient summer temperature and land conditions (e.g. vegetation cover) provide deterministic controls. Warmer temperatures in summer kick off bacterial activity, and in turn, biogenic activity with availability of moisture and ‘key’ nutrients from snow-melt and atmosphere. As the nutrient-inventory of soils at higher altitude could be highly limited and dependent on ambient environmental conditions, soil-biology switches itself in a highly self-adaptive mode to uptake nutrients from atmospher, for example by converting atmospheric N_2_ to reactive/utilizable N via nitrogenase bacterial activity^26^. Over eastern Himalaya, nitrogenase activity has been reported to be highest in month of July and lowest in December, when cold and dry conditions cease/impede nitrogenase activity, while warmer summer months support it^27^. Therefore, at Kedarnath, the bulk of the soil organic matter (with carbon and nitrogen as major constituents) must be produced *in-situ* during summer (warmer) and wet (monsoon) conditions. Such a scenario would favour sedimentary δ ^15^N values to ~0.0‰, as the atmospheric N_2_ pool is assumed to have δ ^15^N values of 0.0‰^28^. Thus, enhanced sediment total nitrogen (TN) concentrations would be associated with lower δ ^15^N values indicating warmer and wetter conditions (intensified ISM), and vice-versa. A few samples collected for assessing inorganic carbon (carbonate carbon) contents were found to be low in concentration (<1%). This the sedimentary total carbon (TC) actually represents total organic carbon (TOC) content of peat sections. A statistically significant correlation found between sedimentary TC and TN depth-profiles of the studied-sequence (r^2^ = 0.88; p < 0.01; n = 122) supports aforementioned contention and also corroborates the fact that bulk of the TC (or TOC) was produced *in-situ* during environmental conditions favouring atmospheric N_2_ fixation. Following this analogy, warmer and wetter phases of the ISM would be associated with lower δ ^15^N values and higher TC/TN contents. Conversely, colder and dry conditions (weaker ISM) would be characterized by relatively higher δ ^15^N values and lower TC/TN contents in peat-sediment layers.

The TC δ ^13^C values (if TC ≈ TOC) generally mimic prevalent vegetation types (C3, C4, or CAM) and atmospheric uptake of CO_2_ through photosynthesis^29^. In general, δ ^13^C values for C3- type vegetation ranges between −25 to −30‰ and −10 to −15‰ for C4- type vegetation^30^. At this high-altitude region, the peat deposition activity could be mainly driven by surface biogenic activity *i.e*. photosynthetically induced carbon sequestraion channelized via atmospheric N fixation. During pre-industrial times, inter-hemispheric differences in atmospheric CO_2_ conentrations were minimal (~1 ppm out of 280 ppm)^31^. Further, the Taylor Dome ice core data reveals about a 25 ppm increase in ambient CO_2_ levels with very little variability in δ ^13^C of CO_2_(−6.5±0.13‰)^32^ between ~8.2 ka BP to ~1 ka BP^32^. Changes in terrestrial biomass have been hypothesized as major cause of variations in pre-industrial atmospheric CO_2_. Hence, observed TC (and δ ^13^C) variability in the peat layers could be utilized to determine senstivity of terrestrial biomass with respect to changing enviornmental conditions in the tropics. In this scenario, warmer and wetter conditions would tend to be associated with enhanced biogenic activity (Carbon sequestration) at high-altitudes of Himalaya (higher TC and δ ^13^C values), and vice-versa. A similar situation (*i.e*. enhanced biogenic activity and fertile soil cover) could be anticipated over generally infertile (snow-covered) landmass around Polar Regions.

At this altitude, cold and dry periods are associated with alternate frosting and thawing events resulting in higher physical weathering of rocks of the catchment, and enhancing the supply of clastics to basin. Since the catchment is predominantly composed of granite and crystallines (rich in iron-bearing minerals), higher clastic supply would lead to the increased magnetic susceptibility of the sediment (sediment-χlf) and higher abundance of major crustal elements (*e.g*., Al, Fe, etc.) and vice-versa. Thus, enhanced (declined) values of sediment-χlf should indicate colder (warmer) phases of climate. Statistically significant correlation was found between sediment-χlf and δ ^15^N depth-profiles (Pearson correlation r = 0.63; and spermann rank correlation (non-parametric) ρ = 0.58; p < 0.01; n = 122) support the aforementioned contention.

**Figure 2:**
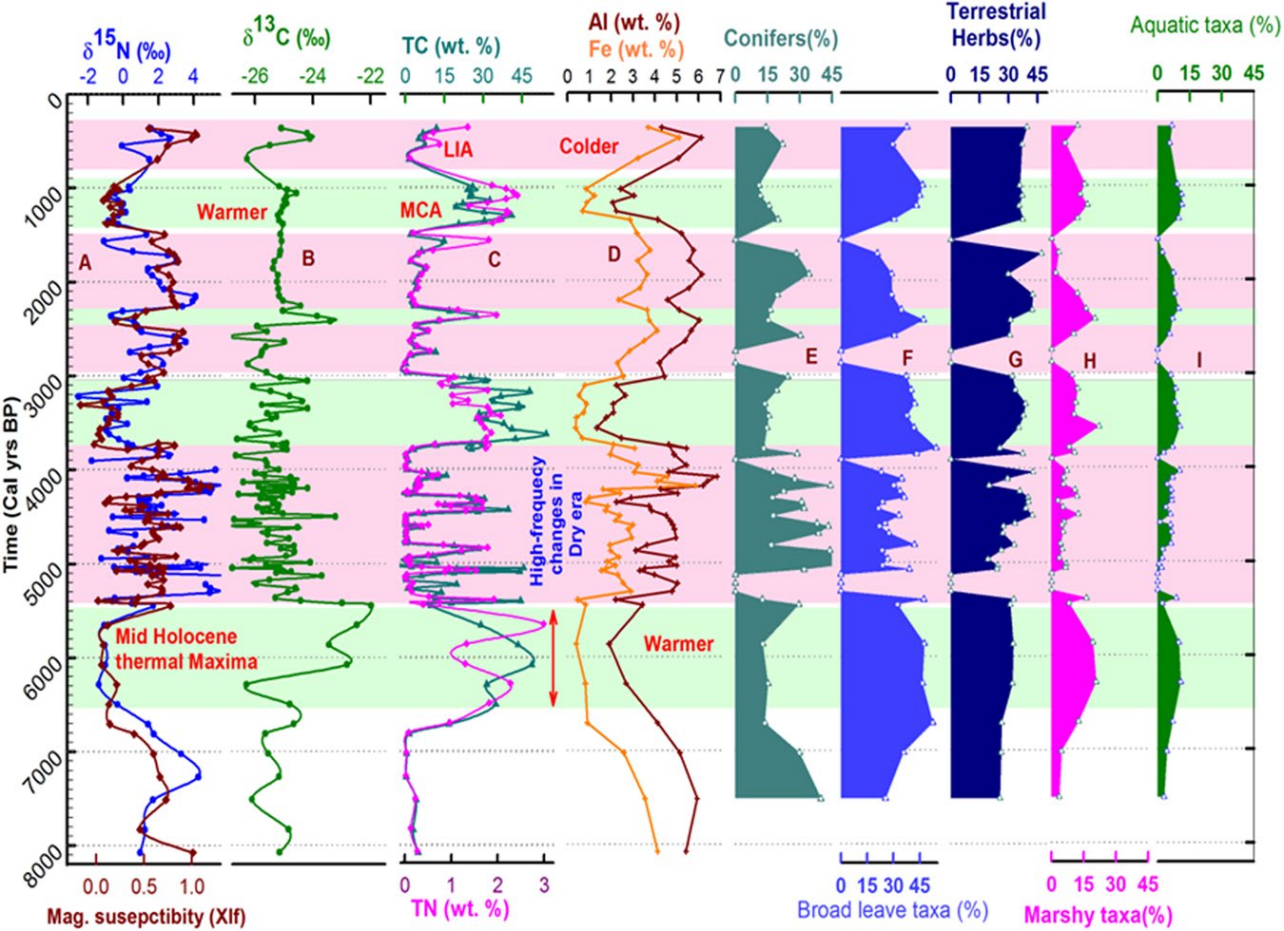
Depth profiles of sedimentary section (**A**) δ ^15^N and magnetic susceptibility (χlf), (**B**) δ ^13^C, (**C**) TC and TN weight fractions; (**D**) Weight fractions of crustal elements Fe, Al; (**E**–**I**) depth profiles of pollen assemblages. Higher TC, TN contents, lower δ ^15^N and χlf represent warmer periods (viz. medieval warm period MWP; shown by green coloured horizontal bands) supporting atmospheric N fixation, while colder periods (viz. Little Ice Age LIA; shown by pink coloured horizontal bands) are dominated by higher χlf, δ ^15^N, crustal element abundance. Pollen assemblage patterns collectively corroborate aforesaid contention (see text for details).

Examination of the pollen assemblage helps to validate the developed interpretative framework described above. The pollen assemblage was analysed and grouped under two categories: (i) arboreal (trees and shrubs) and (ii) non-arboreals (terrestrial herbs, Marshy herbs and aquatic taxa). Among the trees, themorphilic (warmth loving) broad leave taxa *e.g*. *Alnus*, *Betula*, *Rhododendron* and *Quercussemicarpifolia*, and conifers (*Pinus*, *Abies*, *Picea*), are the major components, whereas, terrestrial herbs constitute Poaceae and other steppe vegetation taxa. Marshy elements incorporate taxa that are derived from steppe and meadow vegetation with aquatic elements (Fig. 2E–I). The relative increase in thermophilic broad leaf taxa over conifers and enhancement in marshy and aquatic elements suggest moist conditions (intense ISM activity periods)^15,33,34^. Improvement in conifers and decline in marshy and aquatic elements indicate colder conditions such as during the lower ISM periods^35^. The vegetation mosaic around Kedarnath which results from the varying topographic (altitudinal ranges, slope gradient), climatic (rainfall pattern, wind circulation) and edaphic factors, is dominated by conifers (~50%)^23^. Sub-alpine vegetation occurs below the elevation of ~3500 m AMSL^36^.The forest vegetation (arboreal) in the region is composed of broadleaf and conifer tree, taxa namely *Abies, Cedrus, Cupressus, Juglans, Juniperus, Myrica, Pinus, Quercus Rhododendron, Acer, Betula, Alnus *and* Taxus*,which grows up to ~3200 m AMSL. Among these, *Pinus* and *Quercus* are the major constituents. The important shrubby elements are *Artemisia, Berberis, Cotoneaster*, and *Ephedra*. The meadow type of vegetation is found to occur in the alpine zone above 3500 m AMSL and is represented by *Aconitum heterophyllum, Asteralbescenes, Potintilla, Primula*and other elements of members of Apiaceae, Asteraceae, Brassicacae, Convolvuaceae, Chenopodiaceae/Amarabthaceae, Fabaceae, Polygonaceae, Papaveraceae, Primulaceae, Rosaceae, Rutaceae and Saxifragceae families^37,38^.

The dominance of arboreal taxa along with alpine meadow herbaceous taxa in the peat-sequence suggests that the depositional site was not very far from the tree-line. Pollens of conifers, broad leaf taxa and shrubby elements were transported by the upthermal winds from lower elevations. In the Kedarnath region, the pollen of *Pinus* has high abundance amongst the arboreals^23^ because pollens of *Pinus* are produced in a large quantity as compared to other conifers, and they also get transported large distances by winds. This vegetation milieu also points towards warm and moist climate conditions and expansion/shifting of floral belts towards higher altitudes. Herbaceous vegetation along with marshy and aquatic elements growing in the local surroundings of the peat bog were deposited. Thus, the catchment area for arboreal pollens was likely in the vicinity of the site, as evident by the fact also that, gradient of mountain slopes are very steep, and herbaceous and aquatic pollens were locally derived.

## Results

The proxy-data is tabulated and provided in the supplementary information (Tables S2, S3). Depth profiles of δ ^15^N, χlf, δ ^13^C, TC, TN, Fe, and Al are shown in Fig. 2A–D. Abundances of characteristic pollen-assemblages are also shown in the Fig. 2 (panels E&F). The δ ^15^N values of most of the peat-section lie between −2.5 to 1.0‰, a typical range exhibited by soils that fix atmospheric N_2_^39^. Excellent correlations were found between TC and TN concentrations (Pearson correlation r = 0.82; spearman rank correlation (non-parametric) ρ = 0.90; p = 0.01; n = 129) implying direct link between the organic productivity and nitrogen loading. Statistically significant negative correlation observed between TN contents and δ ^15^N values (Pearson correlation r = −0.490 and spearman rank correlation (non-parametric) ρ = −0.603; p = 0.01; n = 129) suggest dominance of atmospheric N_2_ fixation for peat-sediment organic matter productivity^40^.

Elemental concentrations of Al and Fe in peat-sediment layers were strongly correlated with each other (Pearson correlation r =  0.911 and spearman rank correlation (non-parametric) ρ =  0.923; p = 0.01; n = 65); and this is apparent from their co-varying depth-profiles (Fig. 2D). Fe/Al ratios had a statistically significant positive correlation with sediment-χlf values (r = 0.63; p = 0.01; n = 65), suggesting enriched magnetic mineral concentrations in clastic-supply component of sediment layers throughout.

All the proxies (δ ^15^N, χlf, δ ^13^C, TC, TN, Fe, and Al) follow nicely the interpretative framework developed earlier throughout the deposition period in the last ~8000 years (Fig. 2) suggesting alternating periods of strengthened and weakened ISM. Proxy variations during the last ~1200 year appear to present the contrasting monsoonal conditions. Between ~600−250 calyr BP, Fig. 2 shows reduced TC, TN contents, enhanced Fe and Al contents, enhanced sediment magnetic susceptibility (χlf)and the reduced abundance of broad leaved, marshy and aquatic taxa. All these indicators collectively hint colder conditions and the suppressed monsoonal activity. Relatively enhanced δ ^15^N values during this period corroborate further a lack of atmospheric N input via N fixation, hence implying the prevailing frozen condition. This time-span closely overlaps with globally-recognized cold climatic phase known as Little Ice Age^41^.

In contrast, the preceding warmer period between ~1250 to ~800 Cal yr BP is known as the Medieval Climatic Anomaly (MCA)^41^ and a complete reversal in all the measured proxies was observed (Fig. 2), suggesting a warmer and wetter phase of climate. Further, our data reveals that monsoonal climate was, in general, dryer and colder between ~1400 to ~3000 cal yr BP, except for a short time-window ~2300−2500 cal yr BP, during which a wetter and warm climate appears. Prior to this dry epoch, ISM intensity appears to have been stronger between ~3,800−3000 cal yr BP(Fig. 2).Further, between ~3,800 to 5,400 cal yr BP, the chemical, magnetic, isotopic, and pollen proxies collectively indicate high-frequency variability indicative of a variable climate (Fig. 2A–F). During this phase, δ ^15^N values varied between −2‰ to +4‰indicating climate as oscillating between warm (suitable for atmospheric N fixation) and nearly frozen conditions. Overall, this period could be termed a dryer and colder epoch, with frequent occurrence of warmer and wetter intervals (Fig. 2).

The high-frequency climate change era was preceded by an overall warmer and wetter phase of ISM, considered here as the mid-Holocene climate optimum, which is a well-recognized climatic epoch that prevailed most likely just after sea levels stabilized globally following interglacial melting^42,43^.

### Regional Correlations

Our reconstructed continuous monsoonal-climate record for the last ~8000 calyr is probably the first from the Indian sub-continent that appears to have captured sharp changes in operative forcing factors and feedback mechanistic links. The complexity necessitates contextualization with other contemporary climate records. Salient features of the inferred ISM variability from the Kedarnath area spanning the last ~8000 years indicate the following: (i) warmer and wetter conditions overall during the mid-Holocene (~6500 to ~5400 calyr BP); (ii) high-frequency variability between ~5,400 to ~3,800 calyr BP and an overall dryness; (iii) stronger (weaker) phases of ISM between ~3,800 to 3000 calyr BP (~3000 to 1400 calyr BP); and (iv) conspicuous evidence of wet-warm (MCA) and cold and dry (LIA) phases of climate. Such variability in climate/ISM has not been evidenced yet in any other continental palaeo-climate record for India. Holocene ISM variability from the western Arabian Sea (Hole 723A) determined from an abundance pattern of planktonic foraminifera *(G. Bulloides*) typically follows northern hemispheric summer insolation peaking ~9 ka BP followed by a general declining trend with a weaker sub-millennial scale variability^44^. In contrast, continental records do not generally reveal such a smooth evolution of the ISM. Lake records from western and central India show moist to dry reversal of the ISM during the early to mid-Holocene^45^. The Lonar Crator Lake record from central India reveals alternating wetter and dryer phases, but reiteration of the wetter phase associated with the ISM ~8 to ~5.5 ka BP^46^. The Tso Kar Lake record and the peat record from Chandra Tal from the Ladakh region which received rainfall due to both ISM and westerly winds also implies a major transition phase from wetter to dryer monsoonal climate during the mid-Holocene^13,15^. Likewise, the speleothem-record of Mawmluh Cave in northeast India shows an overall wetter phase of ISM during ~8 to ~6.5 ka BP, a declining monsoon phase between ~6.5 to ~5.4 ka BP, and a relatively dry ISM-phase between ~5.4 to ~4.1 ka BP.A reversal to wetter climate occurs thereafter^47^.

The Lonar Crator Lake record, Central India, shows a drier MCA and ameliorated LIA^46^. Similar conditions are reported further north in pollen based records of the lakes of Ganga Plain^48,49^. Lakes in Rajasthan in western India showed an episodic wet and dry climate^45^. Rivers in arid western India experienced higher flood-frequency during MCA and reduced frequency during the LIA^50^. In Tso Kar Lake of Ladakh, the pollen based studies report wet and cool phases during this time but their temporal correspondence with LIA and MCA could not be resolved, whereas these events are well-recorded in the peat records from Chandra Valley (south of Tso Kar Lake)^15^. A speleothem record from the Kumaun Lesser Himalaya suggests a wetter LIA in the lower reaches of the Himalaya^51–53^. However the rivers in Himalaya experienced lower flood frequency during the LIA and increased flood frequency during the MCA^5,53^. Records from the eastern Arabian Sea have indicated reduced upwelling and low monsoonal activity over the Indian sub-continent during the LIA and strengthening during the MCA^8,44,54^. Thus the evidence for LIA and MCA from the Kedarnath area is not spatio-temporally coherent with the palaeo-climate of the ISM core region. However, our present study from higher reaches of the Himalaya (Kedarnath and Chandra Tal) and studies from the Arabian Sea present a coherent picture of ISM during the MCA-LIA transition. The inconsistent (wetter conditions) LIA from Kumaun-Himalaya may be due to orographic influence. This factor may be explored much more in future, however the Kedarnath area lying on the periphery of the ISM, appears to be more sensitive to the primary force that governed ISM variability during the order of LIA and MCA. This will be better demonstrated in the next section, where we contextualize ISM variability recorded at Kedarnath area with remotely located contemporary proxy records. Including the Kedarnath peat-record, regional Holocene ISM variability is pictorially presented in Fig. S4, which suggests a decent degree of regional coherence among different proxy records considering different proxy behaviour, chronological constraints, temporal resolution, and above all the ‘geographical variability of the ISM’ in this vast tropical region that is appreciable even in the modern instrumental era.

The regional comparative picture (Fig. S4) also reveals that the recovered ISM record from the Kedarnath region is unique in terms of its continuity; and it captures sharp changes especially during the mid-Holocene. A variety of proxy climate records from the north Atlantic, Africa, and tropical Pacific have revealed sub-century/multi-decadal scale variability in global climate since the mid-Holocene. In view of this fact, we examine the Kedarnath ISM record in light of key global climate records of the last ~8 ka BP in the next section.

We judiciously selected and investigated proxy records recovered from across the globe. For instance the ISM is known to be considerably modulated by the El-Nino Southern Oscillation (ENSO), the Indian Ocean Dipole (IOD), and thermal conditions over the Tibetan Plateau^55–57^. The ISM and ENSO linkage(s) have strong bearing on developing more realistic models for long-term ISM variability^46^. Influence of the already recognized controlling factors during low rainfall phases such as the Indian Ocean Dipole (IOD) and north Atlantic sea surface temperatures (SSTs) could be better understood if we can recognize a pattern of ISM variability recorded at Kedarnath in relation to global Holocene climate evolution. We, therefore, explore plausible tele-connections in the Kedarnath ISM record with northern hemispheric climate changes recorded at the polar region (Greenland), long-term of ENSO scale variability from Peru and Peru margin, and century scale variability recorded in the north Atlantic, in terms of ‘Bond’ events.

### Tele-connections between Kedarnath ISM record and global climate indices

#### Synchronicity between ISM and northern hemispheric temperature variability since mid-Holocene

**Figure 3:**
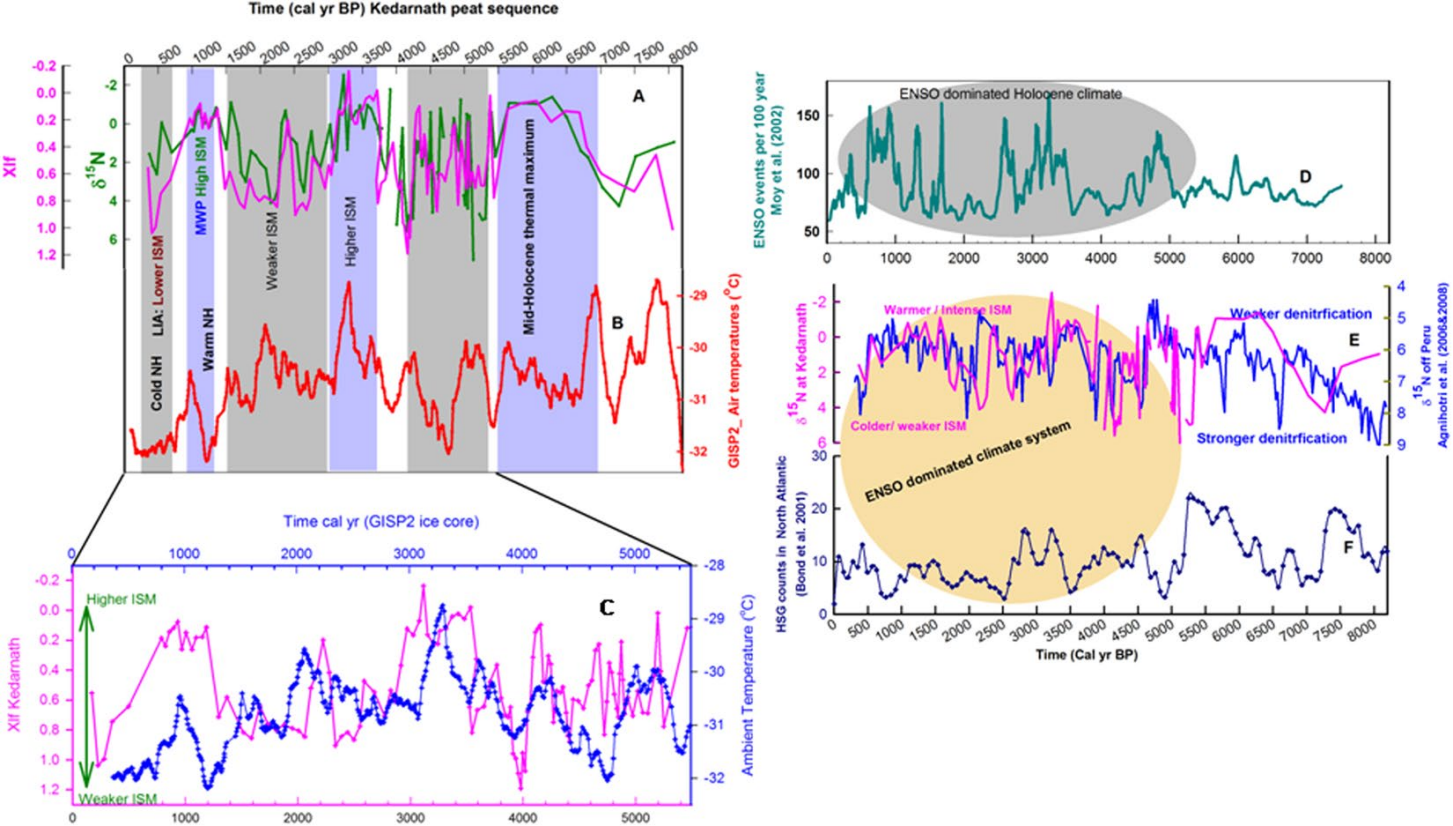
Comparison of Kedarnath’s proxy-based ISM variability with contemporary palaeo-climatic records recovered from diverse geographic locations. (**A**) δ^15^N and magnetic susceptibility (χlf) records show compelling resemblance with (**B**) air-temperature variability recorded at GISP2 ice core at Greenland. (**C**) Similarity between the two records is more conspicuous since mid-Holocene (~5500 yr BP), where a −200 yr a constant offset was applied to age-depth model of Kedarnath peat sequence. (**D)** ENSO events per 100 year recorded in a Peruvian-lake showing dominance of ENSO system since mid-Holocene. (**E**) Covariance between d ^15^N patterns of Kedarnath peat-sequence and those recorded at Peru-margin (Agnihotri *et al*., 2006), noticeable arrival of high- frequency oscillations since mid-Holocene. (**F**) Hematite stained grains (HSG) counts in north Atlantic showing ‘Bond events’ indicating a significant change in the system at ~5.5 ka BP.

**Figure 4:**
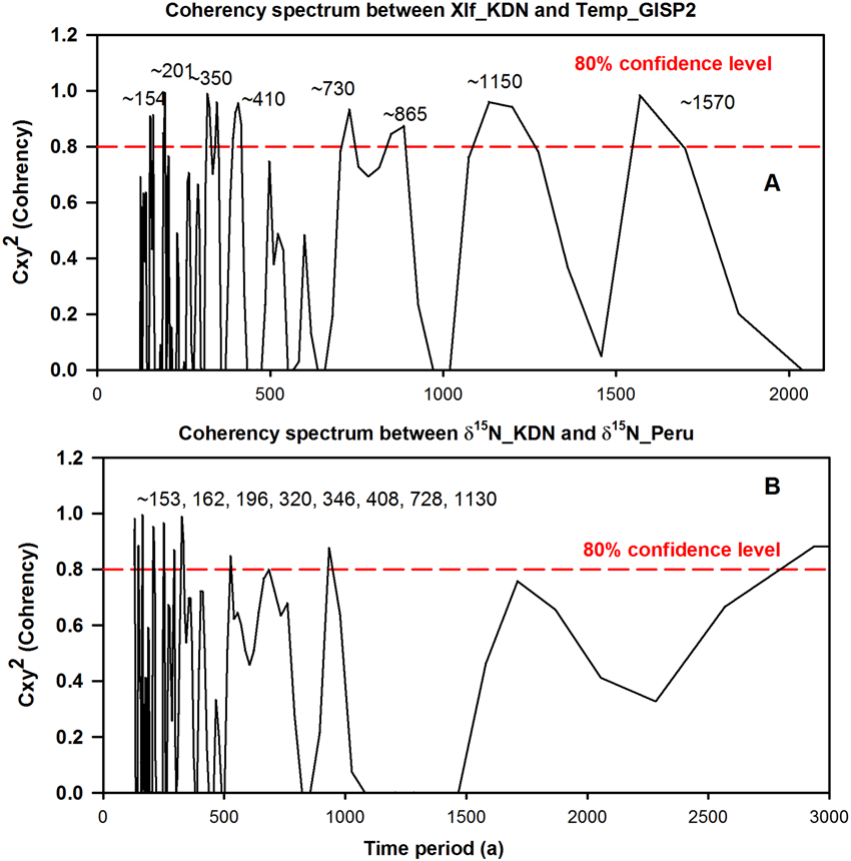
Panel ‘A’ shows cross-coherency spectrum computed between χlf time-series data of Kedarnath peat-sequence with air-temperature data recorded in GISP2 ice core from Greenland (a −200 yr offset applied to the age-depth model of Kedarnath peat-sequence). Panel ‘B’ be shows cross-coherence between δ ^15^N records of Kedarnath peat-sequence and that of Peru margin (a +230 yr offset applied to the age-depth model of Peru δ ^15^N record). Cross-coherency spectral analysis was carried out using SPECTRUM program (Schulz & Stagger, 1997) designed for handling unevenly spaced palaeo-climatic time-series data. Significant coherencies (>80% confidence level) have been shown in respective panels. (See text for detailed interpretation).

The tropical monsoon system has been recognized as one of the important components of the global climate system^58^. Thus, natural ISM variability and its sensitivity against operative forcing factors is key to evaluating response of the global climate system to concurrent anthropogenic warming^59^. Thus, it is important to examine the ISM variability we have reconstructed for the last ~8000 years with that of the northern hemispheric (NH). The NH climate variability is believed to be well-preserved in ice cores of Greenland (GISP2 ice core).Time-series data for inferred ambient temperature changes are available in the public domain^60^. We compared our derived ISM variability for the Kedarnath area with temperature changes recorded in the GISP2 ice cores. Figure 3 shows depth-profiles of δ ^15^N and χlf along with temperature changes recorded in ice core GISP2 for common period. For the last ~5500 years (shown in the Fig. 3C), it can be seen that all warmer epochs recorded at GISP2 appear to be coincident with warmer/phases of ISM especially for last ~5500 yr BP. Importantly (i) sediment δ ^15^N, although a traditional biological proxy in lacustrine and marine environments, is being used here as a sensitive temperature proxy for the Kedarnath region; and (ii) time-series data of two palaeo-records have their independent chronologies, and therefore, different temporal resolutions. If we apply a −200 yr constant offset to the age-depth model of Kedarnath peat sequence, the similarity between the two (tropical and polar) climate records is more obvious for the last ~5500 years (see Fig. 3C). It can be clearly seen that ambient temperatures have risen between ~4.8 to 3.2 ka BP in step-wise fashion so as has ISM intensity. Likewise, from ~3.2 to ~1.2 ka BP, there is again a similar decline. Finally a warm and wetter ISM phase from ~1.2 to 0.7 ka BP (overlapping with MCA) find a resembling warmer period in Greenland that peaked at ~1 ka BP (Fig. 3C). Thus, for the last ~5500 years tropical and polar climate appear to show strikingly-similar evolutionary pattern within chronological uncertainties of independent age-models, and re-stress dominance of tropical climate over global climate since mid-Holocene^61^. Statistical coherency (Cxy) between sediment χlf data and air-temperature data recorded at GISP2 ice core was computed using a spectral analysis program designed especially for unevenly spaced time-series data^62^. Figure 4A clearly demonstrates that two unevenly spaced proxy based time-series data show several statistically significant (>80% confidence level) coherencies. Important to remember that upwelling based monsoonal records from the western Arabian Sea have shown strong linkages with NH climate records for late-Quaternary period^63^.

#### ISM variability at Kedarnath and El Nino Southern Oscillation (ENSO) dominated climate recorded off Peru

The ENSO phenomenon of tropical Pacific is known to exert the most prominent ‘natural’ climate- oscillations on inter-annual timescale and provide a major control over ISM variability, as evident in the modern instrumental recording era. Though the Kedarnath peat record is of a ~50 year resolution, it could be interesting to examine inferred ISM variability in light of long-term ENSO dominated climate records. It is important to note that, in climate records, ENSO oscillations are believed to have arrived since the mid-Holocene between ~6−5.5 ka BP^64–66^. The ENSO events per 100 years clearly reveal significant enhancement from ~5000 calyr BP^64^ (Fig. 3D). Figure 3E shows depth-profiles of sedimentary δ ^15^N of sediment cores recovered from the Peru margin area together with sediment δ ^15^N of peat- layers from Kedarnath area. Both the sedimentary records have independent age-models and temporal resolutions, yet the marine sedimentary record of δ ^15^N off Peru shows compelling covariance with peat-layer δ ^15^N variability captured in Kedarnath area, if we apply a constant +230 year offset to the age-depth model of Peru margin sedimentary record (Fig. 3E). Hence within overall temporal uncertainties involved with retrieved sediment δ ^15^N records from Kedarnath area and Peru margin (maximum ~200–250 years), similar co-varying patterns mimicking ISM variability over tropics of NH and multi-decadal ENSO variability recorded in Peru margin sediment is intriguing and noteworthy for understanding dynamical tropical climate variability across the equator. Again, using spectral analysis program designed especially for unevenly spaced time-series data^62^, we computed statistical coherency (Cxy) between the two proxy-records. As can be seen in Fig. 4B, two δ ^15^N records show several significant (>80% confidence level) periods of coherency (Coherency computed by SPECRUM software is shown supplementary Fig. S5). Due to associated uncertainties with age-models it is difficult for us to assess lead/lag relationship if any. However, our cross-coherency evaluations clearly indicate existence of tele-connected climatic manifestations across the tropics from sub-surface waters of Peru coast in southern hemisphere to high reaches of Himalaya (northern hemisphere). Instrumentally measured time-series meteorological data having annual resolution could be used to explore underlying physical mechanistic links. Palaeo-data retrieved in our study thus could be highly useful to identify such climate hot-spots which could be exerting profound impact on natural climate variability. From the aforementioned synchronized tropical climate records (from mid-Holocene) it appears that, ENSO type variability in south tropical Pacific dominates during warmer and intense ISM epochs, which could be understood in light of observations from the modern instrumental era which reveal that El Nino and La Nina are likely after a good and poor monsoon year respectively.

In order to contextualize obtained ISM record of Kedarnath in realm of global climate, we also show major cryospheric changes recorded in the north Atlantic (as abundance of Haematite stained grains; HSG; Fig. 3F). A marked shift at ~5000 yr BP could be clearly seen. We surmise it is most likely owing to arrival of ENSO dominated climate era in mid-Holocene. Taken together, all proxy records shown in Fig. 3 strongly indicate that tropical climate system reorganized itself at ~5500 calyr BP and continued up to present times.

In the Indian sub-continent, the mid-Holocene period is known for the arrival of widespread aridity and the advent of several ancient human settlements especially along the Indus- Saraswati river channels in northwest India. Climatic (monsoonal variability) requirements for sustenance and subsistence of contemporary human-cultures is intensely researched and highly debated^67,68^. The Kedarnath peat-sequence based ISM record (Fig. 2) appears to support the school of thought that the Indus (Harappan) human culture developed in a highly uncertain monsoon era^61^.

## Conclusions

The Kedarnath peat-record studied herein provides important clues about past variability of the ISM and ambient climate of India for the last ~8000 cal yrs BP. This unique continental high-resolution, multi-proxy ISM reconstruction has striking similarities with contemporary proxy-records of climate from Greenland to the equatorial south Pacific (Peru margin). Inferred high-frequency ISM variability especially during the mid-Holocene to present climate era is therefore unique and important for evaluating the dominant forcing factors and underlying tele-connections. Sediment δ ^15^N depth profiles from the Kedarnath region and Peru margin reveal a likely coupling between contemporary nitrogen biogeochemistry occurring in sub-surface waters off Peru and high-altitude areas of the northwest Himalaya. This biogeochemical manifestation and its climate impacts must be carefully monitored. This study shows that the higher Himalaya is a sensitive hotspot of global climate on multi-decadal to multi-centennial timescale.

## Electronic supplementary material

Supplementary dataset
