# Supplementary material for: 8000-year monsoonal record from Himalaya revealing reinforcement of tropical and global climate systems since mid-Holocene
Source: Sci Rep. 2017 Nov 6;7:14515. doi: 10.1038/s41598-017-15143-9 (PMC5674060; doi:10.1038/s41598-017-15143-9)

**8000-year monsoonal record from Himalaya revealing reinforcement of tropical and global climate systems since mid-Holocene**

Pradeep Srivastava^1^, Rajesh Agnihotri^2^, Deepti Sharma^3^, Narendra Meena^1^, Y.P Sundriyal^3^, Anju Saxena^2^, Ravi Bhushan^4^, R. Sawlani^5^, Upasana S. Banerji^4^, C. Sharma^5^, P. Bisht^3^, N. Rana^3^, R Jayangondaperumal^1^

^1^Wadia Institute of Himalayan Geology, Dehradun, INDIA;

^2^Birbal Sahni Institute of Paleosciences, Lucknow, INDIA;

^3^HNB Garhwal University, Srinagar, Uttarakhand, INDIA;

^4^Physical Research Laboratory, Ahmadabad, INDIA;

^5^CSIR- National Physical Laboratory, New Delhi, INDIA.

**Supplementary data**

**Material, Methods**

# Peat record and sample collection

The studied peat sequence is composed of total 12 units (Figure 1 and Figure S1). The peat accumulated in the inter-moraine depression between the two moraine ridges. These ridges were dated earlier between 13-7 ka. Laterally the sequence shows lensoid geometry whose thickest part in the middle shows alternating beds of black coloured peat and coarse gravelly organic rich mud. The gravelly beds grade laterally into debris supplied by the hill slope erosion of moraines. The alternating nature of beds indicate that the organic productivity and the peat formation was punctuated by relatively low productivity phases that rather overwhelmed by deposition of hill slope debris. Thus the nature of peat stratigraphy preserves a continuous record of high and low organic productivity that spans Holocene. Here it should be noted that the depression receives moisture from only two sources (i) SW monsoon rainfall and (ii) snow melt; and there exists only single source of sediment *i.e.* pre-existing moraines (Granite and granodiorites). Since the sediment is being supplied from the moraine valley walls it is reasonable to assume that there exists little or no time lag between its erosion and deposition.

From the base, the Unit 1 (~40 cm) contained alternating layers of grey sand and brownish black coloured clayey peat. The Unit 2 (60 cm) comprised black colour clayey peat followed by 50 cm thick Unit 3 composed of black coloured organic mud with sporadic angular gravels. Unit 4 was ~150 cm thick black coloured peat which is overlain by a ~35 cm thick Unit 5 containing blackish grey coloured organic mud with angular gravels. Unit 6 was ~47 cm thick brownish black coloured clayey peat and overlying Unit 7 was ~10 cm thick black coloured gritty mud with sporadic gravels. Unit 8 is ~60 cm thick containing black colour clayey peat. Units 9, 10, 11 and 12 were relatively smaller, ~16, ~24, ~20, ~10 cm, respectively, containing thick black organic mud to brownish fibrous clayey peat with sporadic gravels.

Figure S1: Vertical and Lateral sedimentary architecture of the peat sequence. Note that the (i) sequence is developed in the depression located between the two moraine ridges (ii) Hillslope debris derived from the erosion of moraine is deposited as beds that alternate with peat layers.

**Magnetic Susceptibility**

We measured variety of physico-chemical, palynological and stable isotopic proxies in sub-samples of peat and sand layers. Details of followed methods/ protocols are discussed in Appendix 1. Briefly, rock magnetic properties (mainly magnetic susceptibility χlf data present in this study) were measured using a Bartington MS2B sensor with an overall analytical precision of ~1%^1, 2^.

**Total Carbon, Nitrogen and Isotope measurements**

Sedimentary carbon (C), nitrogen (N) and their isotopic anomalies (δ^13^C and δ^15^N) were measured on isotope ratio mass-spectrometer coupled with an elemental analyzer (EA) in continuous flow mode. The isotopic data are reported using the standard delta notation with respect to Vienna-PDB. Working protocols and standard procedures followed for achieving accurate and precise isotopic measurements using international and *in-house* standards have been described in detail elsewhere (Agnihotri et al., 2014). Briefly, bulk samples were dried, finely powdered, and pelleted in tin cups and then were combusted in EA. Sample were subjected to isotopic analyses without decalcification (as inorganic carbon data generated on few check samples yielded negligibly lower concentrations <1.0 wt.%). Analytical precisions of δ^13^C and δ^15^Ndata were found to be better than 0.15‰ and 0.20‰ respectively.

**Elemental Analysis**

Nearly 0.5 gm of peat-sediment samples were combusted at 650⁰C in furnace to remove the organic matter. Following which the sample aliquots were digested by treating them with concentrated acids (HCl, HF and HNO_3_) with the help of microwave digestion system. The digested samples were dried and dissolved in 2 % HNO_3_ medium. All the reagents used in the present study were of analytical grade. Major element concentrations (Al, Fe, Mg, etc.) were determined in sixty peat- sediment samples (out of total 129 samples) by aspirating the sample solutions in the ICP-AES (JobinYvon 38S) with an overall reproducibility better than ~5%. The reproducibility of the instrument was taken care by using certified reference standard NOVA^3^ and MAG^4-6^.

**Pollen analysis**

Pollen data were generated on 50 samples at different depth intervals using standard procedures. To extract the pollen and spores, 10 gm sediment of the selected samples was boiled with 10% aqueous KOH to deflocculate pollen/spores from the sediment and to dissolve the humic acid. Subsequently, the samples were treated with 40% HF in order to remove silica present in the surface sediments. This is followed by the standard procedure of acetolysis^7, 8^ using acetolysing mixture (9:1 ratio of acetic anhydride and concentrated sulphuric acid) was followed. Finally, the samples for microscopic examination were prepared in 50% glycerine solution.

The precise identification of the recovered pollen/spores in the sediment was carried out by consulting the reference pollen slides available in the sporothek of BSIP Herbarium as well as comparing the pollen photographs in the published literature^9-11^ and available online atlases of pollens ([http://www.ucl.ac.uk/archaeology/about/facilities/archaeobotany/families/poaceae](https://www.researchgate.net/deref/http%3A%2F%2Fwww.ucl.ac.uk%2Farchaeology%2Fabout%2Ffacilities%2Farchaeobotany%2Ffamilies%2Fpoaceae)). The pollen sums range from 310 to 550 pollen grains, depending upon the pollen yield of the samples. Percentage frequencies of the recovered pollen taxa have been calculated in terms of total terrestrial plant pollen. The pollen of aquatic plants and spores of ferns and other lower cryptogams (algal remains) are excluded from the pollen sums because of their origin from the local provenances. The plant taxa classified as trees (conifers and broad leave taxa), shrubs, herbs, ferns and algal remains are arranged in the same sequence in the pollen spectra.

To plot the pollen percentage diagram, the Tilia software (1.7.16 version) was used. To run the depth constrained cluster analysis^12^, on pollen frequencies, CONISS multivariate method using square root transferred data was used and based on the significant changes in the pollen assemblage, four distinct pollen zones have been identified.

**References**

1. Dearing, J., 1994. Environmental magnetic susceptibility. *Using the Bartington MS2 system. Kenilworth, Chi Publ*.
2. Meena, N.K., Maiti, S. and Shrivastava, A. Discrimination between anthropogenic (pollution) and lithogenic magnetic fraction in urban soils (Delhi, India) using environmental magnetism. *Journal of Applied Geophysics* 73 (2), 121-129 (2011).
3. Amin, B.S., Likhite, S.D., Radhakrishnamurty, C.T. and Somayajulu, B.L.K., 1972, March. Susceptibility stratigraphy and paleomagnetism of some deep Pacific Ocean cores. In *Deep Sea Research and Oceanographic Abstracts* (Vol. 19, No. 3, pp. 249-252). Elsevier.
4. Abbey, S. Studies in “Standard samples” of silicate rocks and minerals 1969-1982. *Geol. Surv. of Canda Papers* No. 83-15, 114 (1983).
5. Gladney, E.S. and Roelandt, I. Compilation of boron concentration data for NBS, USGS and CCRMP reference materials. *Geostandards Newsletter* 11(2),167-185 (1987).
6. Govindaraju, K. 1994 compilation of working values and sample description for 383 geostandards. *Geostandards and Geoanalytical Research* 18(S1), 1-158 (1994).
7. Erdtman, G., 1943. An Introduction to Pollen Analysis. Chronica Botanica Co., Waltham, Mass., USA.
8. Faegri, K., Iversen, J., 1989. Textbook of Pollen Analysis, fourth ed. Wiley, Chichester. 328 pp.
9. Chauhan, M.S., Bera, S.K., 1990. Pollen morphological studies of some important tropical plants from Sidhi District, Madhya Pradesh. Geophytology 20 (1), 30-36.
10. Demske, et al., Atlas of pollen, spores and further non-pollen palynomorphs recorded in the glacial-interglacial late Quaternary sediments of Lake Suigetsu, central Japan. Quaternary International. 290-291: 164-238.
11. Nayar, T.S., 1990. Pollen Flora of Maharashtra State. Today and Tomorrow, Printer’s and Publisher’s, New Delhi.
12. Grimm, E.C., 2004. TILIA and TG View 2.0.2. Illinois State Museum, Springfield IL.

**Table S1:** Sample dated using ^14^C contents in organic carbon fractions by accelerator mass spectrometry. See Supplementary Info

**Table S2:** Measured data of TC, TN (wt. %), TC/TN ratios, and sediment δ^13^C and δ^15^N values. See Supplementary Info

**Table S3:** Measured data of sedimentary Al, Fe, Mg (wt.%). (Mg depth-profile is not shown in the Figure 2) See Supplementary Info

Figure S2: The study site (Kedarnath) falls under high annual rainfall domain (shown by upward arrow). The Satellite data used here is 90 m SRTM (Shuttle Radar Topographic Mission; Product courtesy: Unites States Geological Survey) data (https://lta.cr.usgs.gov/SRTM1Arc).


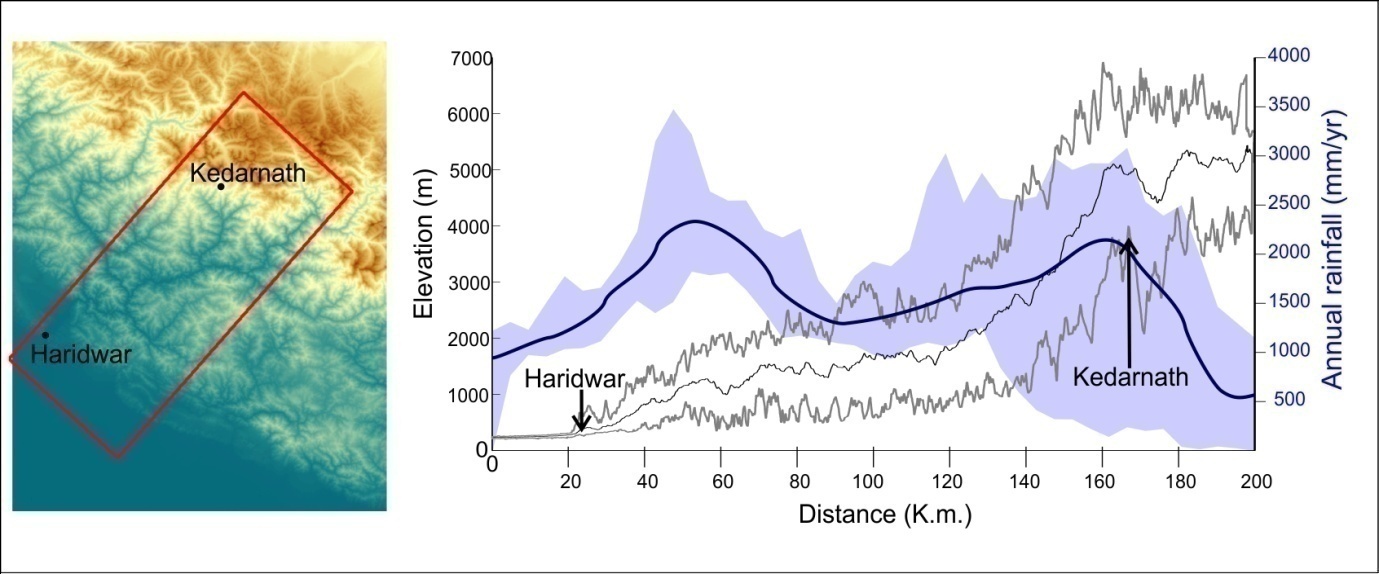


Figure S3: Correlations between All India JJAS rainfall (and annual) and cumulative rainfall over the Uttarakhand state of India. Evidently, strength of correlation for JJAS months is stronger that for annual scale cross-correlation.


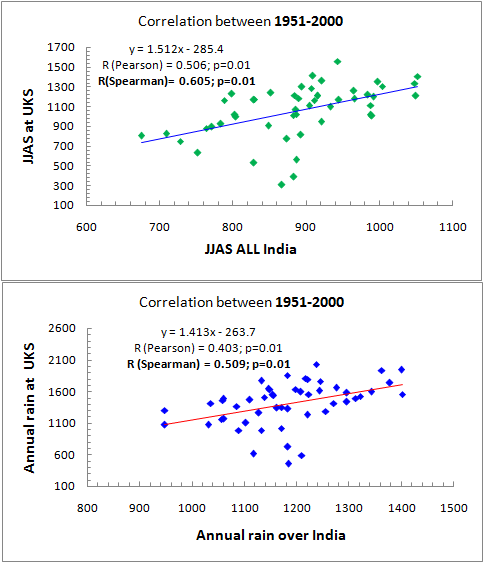


Figure S4. Comparison of inferred Holocene ISM variability recovered from various proxy records with the Kedarnath record. (A) Wetness record from Lunkaransar lake (Thar Desert, India (Enzel et al., 1999) (B) Lonar lake record from central India (Prasad et al., 2014) (C) Tso Kar lake record from Laddakh (India) (Wünnemann et a., 2010) (D) Speleothem record from Mawmluh cave (northeast India) (Berkelhammer et al., 2012 (E) variations in abundance *of G. bulloides* in Hole 723A from western Arabian Sea (Gupta et al., 2003) and (F) Present Study.


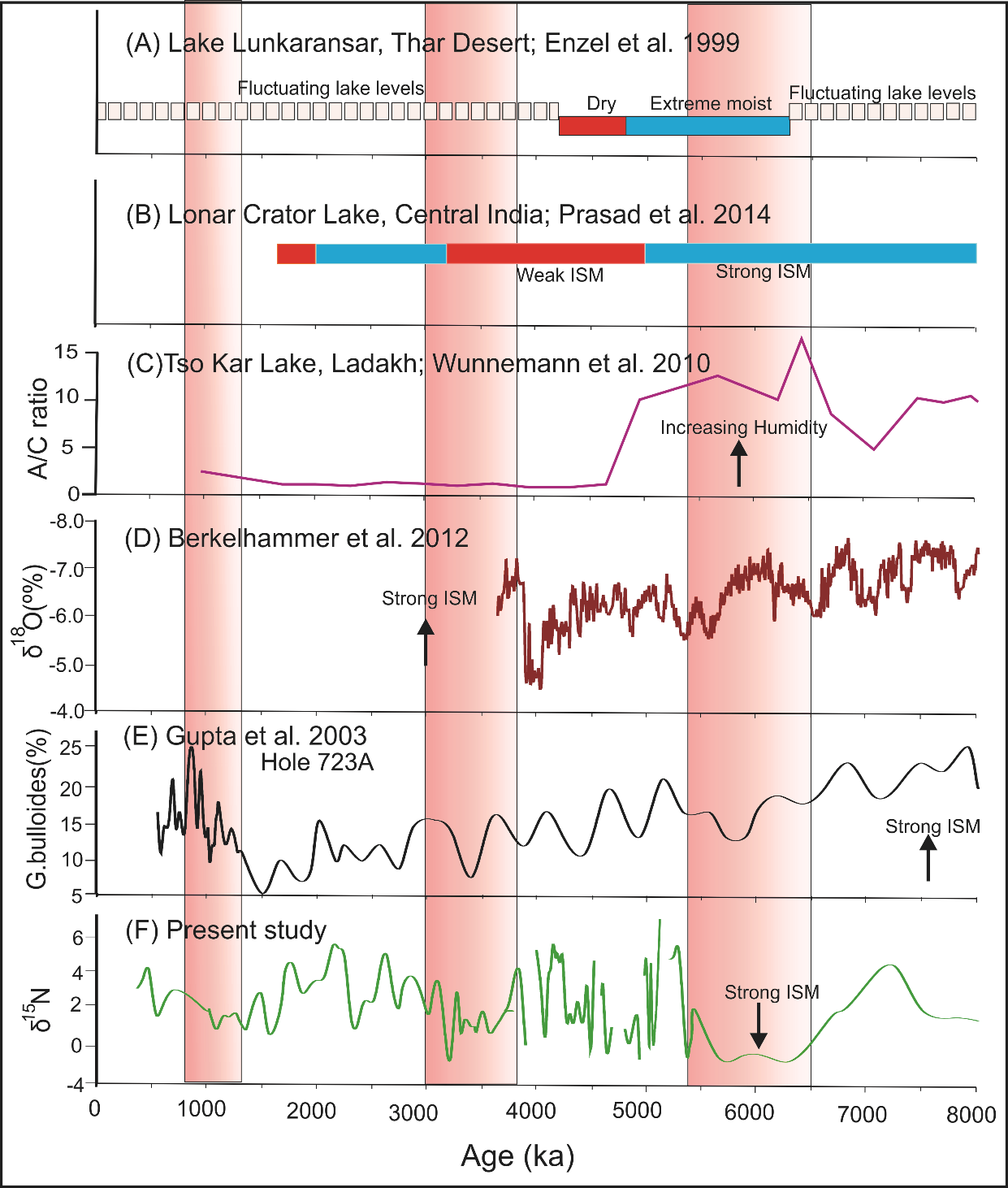


Figure S5: Cross-coherency spectrum of Kedanath sediment χlf (proxy for ISM) and air-temperature variability deduced from GISP2 ice core from Greenland. The cross-coherency was computed by applying −200 yr a constant offset to the age-depth model of Kedarnath peat-sequence (see outset of Fig.3B) without any smoothening of time-series data using SPECTRUM program developed by Schulz and Stagger, (1997). Horizontal line shows significant (at 80% confidence) coherencies at periods denoted by numbers written in the panel.


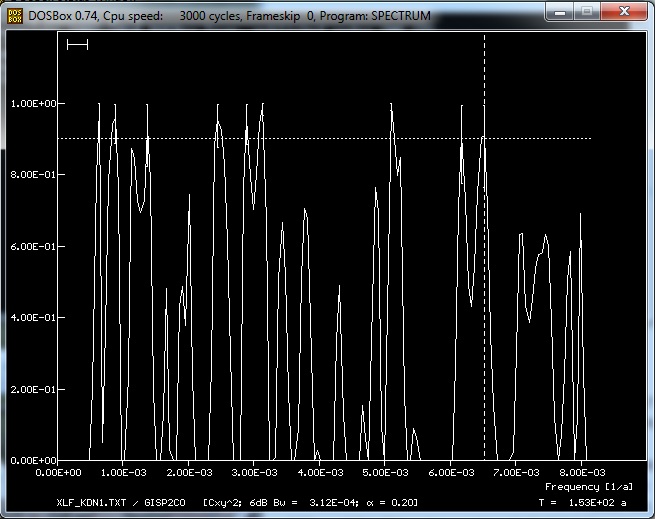

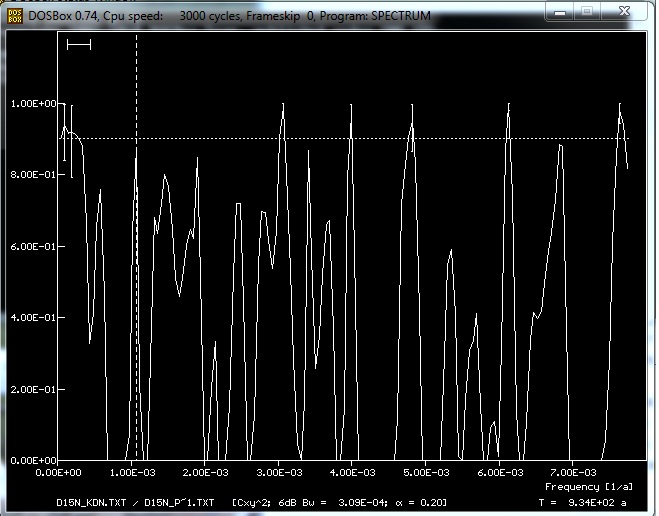

Supplement: Supplementary file 1 — Supplementary dataset [file 41598_2017_15143_MOESM1_ESM.doc]
